# Human Echolocation for Target Detection Is More Accurate With Emissions Containing Higher Spectral Frequencies, and This Is Explained by Echo Intensity

**DOI:** 10.1177/2041669518776984

**Published:** 2018-05-22

**Authors:** L. J. Norman, L. Thaler

**Affiliations:** Department of Psychology, Durham University, Durham, UK

**Keywords:** Audition, cognition, perception, sensory plasticity/adaptation

## Abstract

Humans can learn to use acoustic echoes to detect and classify objects. Echolocators typically use tongue clicks to induce these echoes, and there is some evidence that higher spectral frequency content of an echolocator’s tongue click is associated with better echolocation performance. This may be explained by the intensity of the echoes. The current study tested experimentally (a) if emissions with higher spectral frequencies lead to better performance for target detection, and (b) if this is mediated by echo intensity. Participants listened to sound recordings that contained an emission and sometimes an echo from an object. The peak spectral frequency of the emission was varied between 3.5 and 4.5 kHz. Participants judged whether they heard the object in these recordings and did the same under conditions in which the intensity of the echoes had been digitally equated. Participants performed better using emissions with higher spectral frequencies, but this advantage was eliminated when the intensity of the echoes was equated. These results demonstrate that emissions with higher spectral frequencies can benefit echolocation performance in conditions where they lead to an increase in echo intensity. The findings suggest that people who train to echolocate should be instructed to make emissions (e.g. mouth clicks) with higher spectral frequency content.

## Introduction

Echolocation describes the process by which an organism perceives their external environment through reflected sound waves (Griffin, 1944). Typically, it involves the creation of a sonic emission (e.g. an oral click) by the echolocator that ensonifies the environment (Schörnich, Nagy, & Wiegrebe, 2012), thus inducing the echoes that are reflected back to the echolocator. It is a mode of perception that is most often associated with certain non-human animal species, such as bats and some marine animals (Griffin & Galambos, 1941; Jones, 2005), but it is also well documented (since Supa, Cotzin, & Dallenbach, 1944) that humans are also capable of using echolocation (Kolarik, Cristea, Pardhan, & Moore, 2014; Thaler & Goodale, 2016). Specifically, some humans with vision loss have been known to develop echolocation skills to an exceptional level (e.g. Teng, Puri, & Whitney, 2012), often without formal instruction, and typically use a tongue click as their preferred type of emission to achieve this. Through this, blind individuals are able to access many properties of distal objects in the environment that would otherwise be accessed through vision, such as distance (Schörnich et al., 2012; Tonelli, Brayda, & Gori, 2016), position (Teng et al., 2012; Thaler, Arnott, & Goodale, 2011), size (Teng & Whitney, 2011; Thaler, Wilson, & Gee, 2014), shape (Milne, Goodale, & Thaler, 2014) and material of distal objects (Milne, Goodale, Arnott, Kish, & Thaler, 2005).

One important and practical use of echolocation lies in the detection and localisation of objects in space, as this allows the echolocator to navigate their environment safely (Kolarik, Scarfe, Moore, & Pardhan, 2016). To achieve this, expert echolocators may extract a number of different cues from echoes. As the distance between the echolocator and the target object increases, for example, there is an increase in the delay between the onset of the emission (e.g. mouth click) and that of the echo, as well as a decrease in the overall intensity of the echo. These could be used as cues by an echolocator when judging an object’s distance. It is also possible that the emission and echo sounds will fuse, giving rise to the perceptual experience of a single sound carrying a particular pitch. This fusion may be the result of acoustic interference, that is when emission and echo temporally overlap, or it might be the result of perceptual interference, that is ‘repetition pitch’, where two brief sounds separated by a short gap attain the quality of a single sound carrying a pitch that is inversely related to the duration of the gap (Bilsen, 1966). Cues such as intensity and pitch, however, are not unambiguous in their indication of object distance, as they are also affected by the reflecting object’s size, shape and material. In comparison, when echolocators localise an object in the horizontal plane they may rely on binaural cues such as the interaural level difference of the reflected sound (Rowan et al., 2013; Rowan, Papadopoulos, Edwards, & Allen, 2015).

In order for echolocation to be successfully used as a mode of perception, however, the echolocator must be able to not only interpret the relevant acoustic cues in the echo but also be able to create an emission with effective signal properties. Since the early studies on echolocation, there has been considerable progress in our understanding of the acoustic properties of tongue clicks common among expert echolocators. Mouth clicks of expert echolocators, for example, typically last 3 ms and contain energy at multiple parts of the audible spectrum, with peaks between 2 and 5 kHz and an additional local peak at 10 kHz (de Vos & Hornikx, 2017; Thaler et al., 2017; Zhang et al., 2017). There is also individual variability across echolocators in terms of peak spectral frequency and click duration.

The question arises if there are certain acoustic features of the emission that confer a general performance advantage. Louder emissions, for example, are associated with better performance in detecting changes in room size (Flanagin et al., 2017) and in detecting the presence of a single object (Thaler & Castillo-Serrano, 2016). In contrast, there are some mixed findings regarding the optimum duration of the emission. Rowan et al. (2013), for example, found that object localisation improved with the duration of the emission, and Schenkman and Nilsson (2010) found that object detection improved as the duration of emissions increased (from 5 to 500ms). In comparison, Thaler and Castillo-Serrano (2016) found that shorter emissions were associated with better performance in a detection task. This discrepancy can perhaps be explained by differences in the methodologies of the studies. In Thaler and Castillo-Serrano’s (2016) study, participants used their own mouth clicks to actively detect a real object in a real environment. In the other two studies, however, participants either passively localised an object in virtual acoustic space (Rowan et al., 2013) or passively detected an object in pre-recorded sounds (Schenkman and Nilsson, 2010), in both cases using artificially generated bursts of noise as emissions. The studies also differed in the ranges of emission durations that they used. In Thaler and Castillo-Serrano’s (2016) study, the clicks varied in their duration from 3.4 to 18.1 ms, whereas in Rowan et al.’s (2013) study, the noise bursts varied from 10 to 400 ms, and Schenkman and Nilsson (2010) used noise bursts of 5 ms, 50 ms and 500 ms. It is important, therefore, to determine the extent to which such findings generalise across different types of emission.

Regarding the spectral content of the emission – that is the energy contained within the emission at different frequency ranges within the audible spectrum – it has been observed that individuals who produce clicks containing higher spectral frequencies perform better in tasks of object detection and localisation. Thaler and Castillo-Serrano (2016), for example, found that within a mixed sample of sighted participants naïve to echolocation and two expert echolocators, there was a positive correlation between the peak frequency of the click generated by participants and their performance in a simple object detection task. This correlation, however, was driven by the performance of the two expert echolocators, who both produced clicks with higher spectral frequencies compared to the naïve echolocators. Nonetheless, within a small sample of expert echolocators, two who produced clicks with average peak spectral frequencies of 3.39 and 3.63 kHz, respectively, had smaller perceptual thresholds in an angular discrimination task in comparison to a third expert echolocator whose clicks had a lower average peak spectral frequency of 2.07 kHz (Teng et al., 2012; Thaler et al., 2017;). These results, however, only provide correlational evidence and it remains to be tested experimentally whether clicks containing higher spectral frequencies do in fact lead to better echolocation performance. Rowan et al. (2013, 2017) found that participants’ ability to localise and detect an object in virtual acoustic space was better with noise emissions containing higher spectral frequencies. Specifically, in those studies, participants were more accurate in detecting or localising an object in virtual acoustic space using high-pass (>3 kHz), compared to low-pass (<3 kHz), filtered noise. Yet, they also found that for object azimuth localization, the addition of low frequency sound (i.e. use of broadband noise as compared to high-pass noise only) impaired echo processing (Rowan et al., 2013), while for target detection the addition of low frequency sound actually improved performance (Rowan et al., 2017). Thus, even though there seems to be an advantage of high frequency sound, it is unclear to what degree this advantage persists when low spectral frequency components are present at the same time. Stimuli in these studies were noise bursts, rather than clicks, and echo onset cues had been digitally removed. Thus, it is also unclear whether this result also applies to click emissions typically used by echolocators.

There are potential reasons why an emission containing higher spectral frequencies might be preferable over one containing lower spectral frequencies. Rowan et al. (2013) suggested that the presence of low frequency sound (i.e. < 3 kHz) in the emission may interfere with processing of the returning echo. More generally, however, emissions that contain higher spectral frequencies might simply confer a general advantage for echolocation because they elicit stronger echoes for objects of finite size. Specifically, an object will reflect sound more effectively if the wavelength of the sound is smaller than the object’s size. Given that sounds of higher frequency have shorter wavelengths, it follows that objects of finite size will lead to stronger echoes when the emission contains higher frequencies. This effect is due to both specular and diffraction effects. This might explain the possible advantage of using emissions containing higher frequencies in tasks of object detection.

The first aim of this study was to test the hypothesis that performance in an object detection task using echolocation improves with the use of emissions containing higher spectral frequencies, regardless of whether the emission is a click or noise burst, and regardless of the presence of low frequency sound (i.e. < 3 kHz). If this advantage were found, the second aim was to determine whether it could be eliminated by equating the intensity of the echoes across levels of spectral frequency. If the advantage could be eliminated this way, it would suggest that the underlying cause of the association between spectral frequency content of the emission and echolocation performance lies in differences in echo intensity (at least in tasks of object detection). In order to test this, we first made sound recordings in an anechoic room using a human model manikin. A loudspeaker was fixed to the mouth of the manikin, which emitted artificially generated emissions varying in their peak spectral frequency (3.5, 4.0 and 4.5 kHz). We used both artificial clicks (lasting ∼ 5 ms and modelled after real human mouth clicks) as well as noise bursts (lasting 500 ms) as our emissions. Recordings were made with a wooden disk at 1, 2 or 3 m distance from the loudspeaker or with no object present at all. Sighted participants then listened to these recordings and judged whether or not they heard the reflecting object. They also did the same under conditions in which the sounds had been digitally altered to equate the intensity of echoes at each distance across emissions. We varied the object distance in order to test whether any high frequency advantage generalises to situations where different acoustic cues might be used to detect the object. We used the noise bursts in addition to the clicks in order to determine if the results hold regardless of emission type used.

## Methods

All Procedures followed the British Psychological Society code of practice and the World Medical Association’s Declaration of Helsinki. The experiment had received ethical approval by the Ethics Advisory Sub-Committee in the Department of Psychology at Durham University. All participants gave written informed consent to take part in this study.

### Sound Recordings

#### Emissions

**Figure 1.**
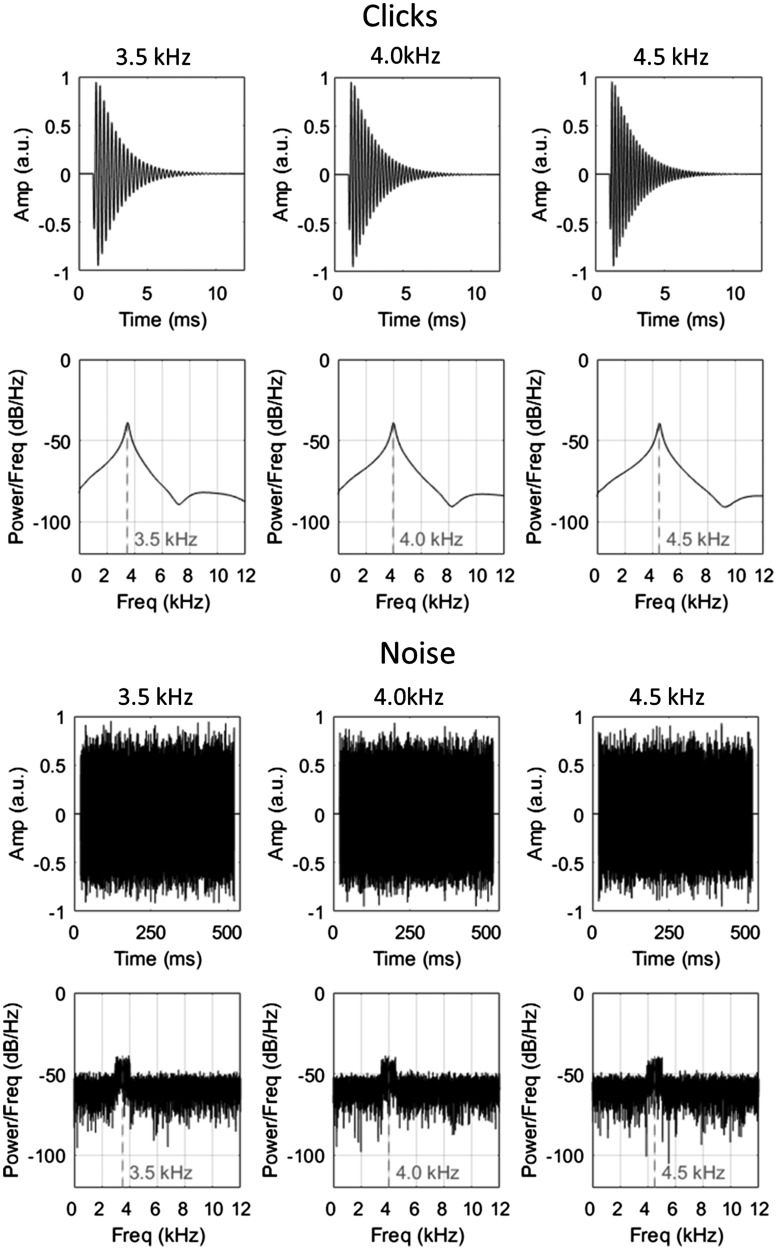
Waveforms and power spectral density functions of the click emissions (upper panels) and noise emissions (lower panels). The clicks were generated by multiplying a sine wave of a particular frequency (3.5, 4.0 or 4.5 kHz) with a decaying exponential. The noise was generated by selectively adding a 9 dB boost (1 kHz passband) to particular frequency components (3.5, 4.0 or 4.5 kHz) of white noise.

The emissions used in the recordings took the form of either a click or noise burst and were artificially generated as wav-files at a sampling rate of 96 kHz and resolution of 24-bit using MATLAB R2015b (The Mathworks, Natick, MA). The click was constructed by first creating a 10 ms sinusoid of the desired frequency (3.5, 4.0 or 4.5 kHz) and then multiplying all values up until the first half period by 0.6. This effectively simulates the rising intensity of a natural click. Then, all values after the first 1.5 periods were multiplied by the output of the decaying exponential function *y* = e^−6^*^x^*, where *x* is a series of linear equally spaced values between 0 and 1 that is equal in length to the number of values in the sinusoid between the first 1.5 periods and its end. This effectively simulates the fall in intensity of a natural click. This type of sound (a sinusoid multiplied by a decaying exponential) has been suggested previously to be a good approximation of the waveform created by a human echolocator’s mouth click (Martinez-Rojas, Hermosilla, Montero, & Espi, 2009; Thaler et al., 2017) and has been used successfully as an artificial emission in previous tasks of echolocation (e.g. Thaler & Castillo-Serrano, 2016). The noise emissions were 500 ms bursts of noise with a 9 dB boost centred at 3.5, 4.0 or 4.5 kHz. The 9 dB boost was created by first filtering white noise with a Kaiser window bandpass filter (1 kHz passband centred at the desired frequency, with 0.1 kHz transition bands either side). This filtered noise was then added to unfiltered white noise, at a relative amplitude ratio of 3:1. The waveforms for all emissions, as well as their spectral power functions, are shown in Figure 1.

#### Apparatus and setup

All sound recordings were made in a sound-insulated and echo-acoustic dampened room (approx. 2.9 m × 4.2 m × 4.9 m) lined with foam wedges (cut-off frequency 315 Hz). Binaural sound recordings were made at a sampling rate of 96 kHz and resolution of 24-bit using a portable digital recorder (Tascam DR-100 MK2, TEAC Corporation, Japan) and in-ear microphones (Bruel & Kjaer model 4101, Denmark). The microphones were placed in the ears of a manikin, the mouth of which was positioned behind a loudspeaker (Fostex FE103En) mounted on a metal pole (1 cm diameter) and used to emit artificially generated sounds. The manikin was custom-made, consisting of a torso and head made of high-density foam covered with soft plastic having a skin like texture. The manikin was constructed based on a commercially available head and torso simulator (BodyRip Punching Dummy Torso), but head shape had been modified to better match anthropometric head and torso measurements (Algazi, Duda, Tompson, & Avendano, 2001). See Table 1 for the model’s head, neck and ear measurements. Furthermore, 5 mm diameter holes had been drilled inside the ears to function as artificial ear canals into which binaural microphones could be placed. Woollen clothing was placed over the torso and a woollen hat was placed on the head to resemble hair. The loudspeaker was driven by a Dell Latitude E7470 laptop (Intel Core i56300U CPU 2.40 GHz, 8 GB RAM, 64-bit Windows 7 Enterprise) through a USB Soundcard (Creative Sound Blaster X-Fi HD Sound Card; Creative Technology Ltd., Creative Labs Ireland, Dublin, Ireland) and amplified by a Kramer 900 N Stereo Power Amplifier (Kramer Electronics Ltd., Jerusalem, Israel). Level of amplification in all electronic equipment was held constant for the recording of all sounds across all conditions.

**Table 1.** Anthropometric Details of the Human Model Manikin Used in the Sound Recordings.

Head width	14.50 cm
Head height	18.50 cm
Head depth	16.00 cm
Neck width	13.50 cm
Neck height	6.50 cm
Neck depth	14.00 cm
Pinna offset down	4.50 cm
Pinna offset back	1.50 cm
Pinna height	6.50 cm
Pinna width	3.75 cm

**Figure 2.**
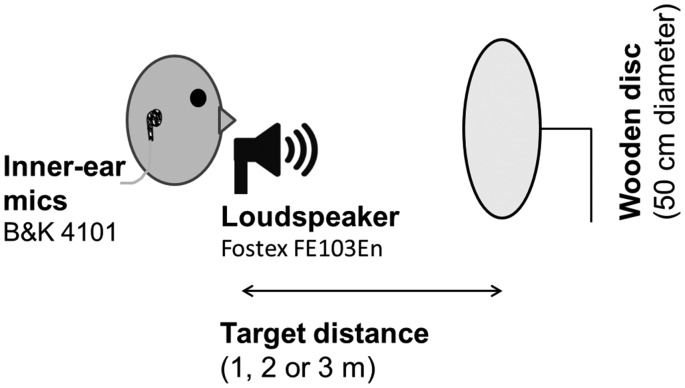
Sketch of the apparatus setup used for making the sounds recordings. A manikin was positioned with their mouth behind a loudspeaker. The loudspeaker emitted either a click or noise burst at one of three major spectral frequencies. A wooden disc was used as a reflecting object and was positioned at a distance of either 1, 2 or 3 m from the loudspeaker, or not present at all. Recordings of the emission and echoes (when the object was present) were made using innerear microphones.

Separate recordings were made with a reflecting object positioned at a distance of 1, 2 or 3 m from the loudspeaker, or with no reflecting object present at all, for each emission type (∼5 ms click, 500 ms noise) and each level of the emission’s spectral frequency (3.5, 4.0, 4.5 kHz). The reflecting object was chosen to be a .8 cm thick wooden disk (50 cm diameter, made from plywood, double coated with matte emulsion paint) also mounted on a metal pole (1 cm diameter) directly facing the loudspeaker, with the height of the disk’s centre matching that of the loudspeaker. The size and shape of the object had been chosen based on previous research in this area (Schenkman & Nilsson, 2010, 2011; Thaler & Castillo-Serrano, 2016) and because this size is relevant to people who use echolocation in everyday life (e.g. to detect the side panel of a bus shelter, a large tree, or a person). The set-up of the recording apparatus is shown in Figure 2. Based on the speed of sound (in air at 20℃), and given the diameter of the reflecting object used, frequencies of 690 Hz (.69 kHz) and below will not return echoes, while higher spectral frequencies will interact with the object and form echoes through specular and diffraction effects. Thus, the object we used in our study will result in echoes from all emissions we used. In this way, the object we used is a ‘worst case’ scenario for testing the effects of emission spectral frequency. Yet, emissions containing higher spectral frequencies are still expected to lead to stronger echoes because of diffraction. Thus, there will be more intense echoes as the peak spectral frequency of the emission is increased from 3.5 to 4.5 kHz.

### Processing the Sound Recordings

**Figure 3.**
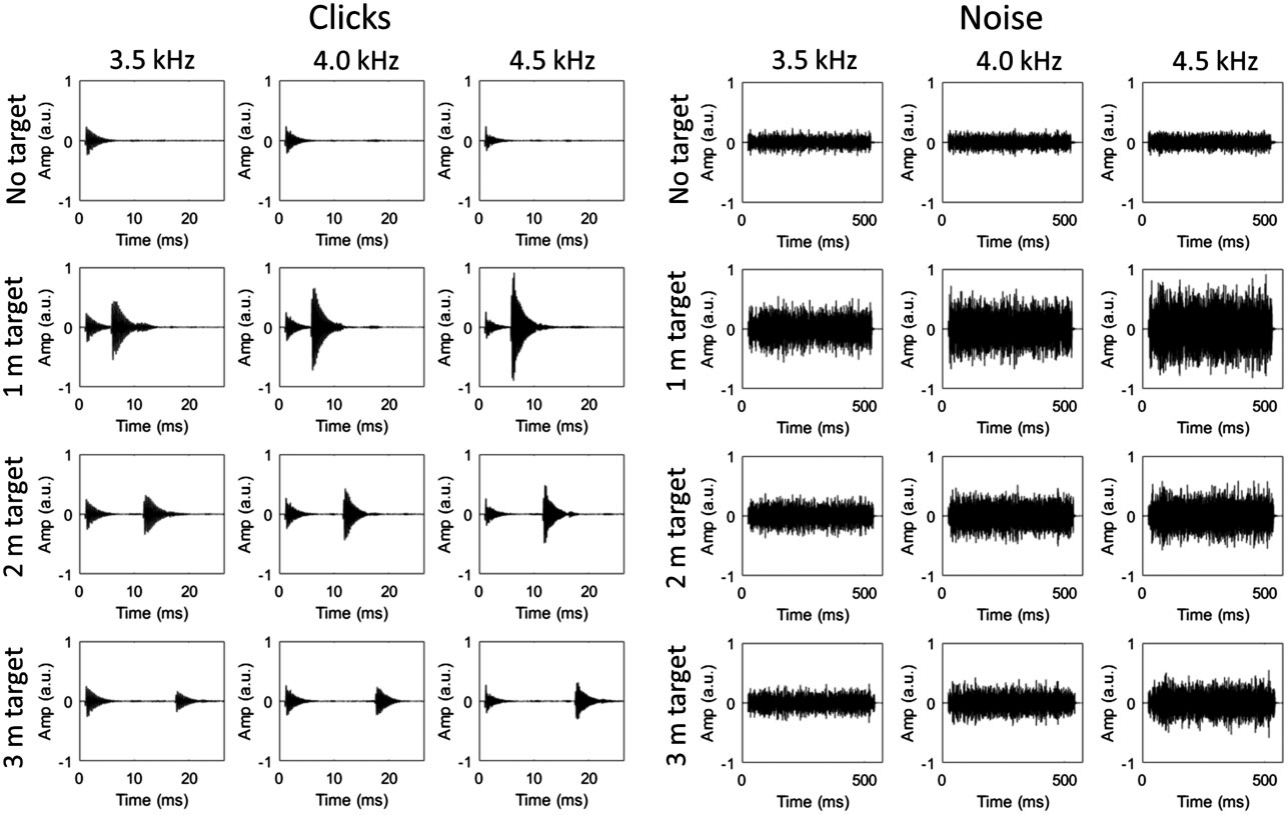
Waveforms of the recorded click and noise emissions. The clicks are shown in the left set of images, and the noise in the right set. From top to bottom: no target, 1 m target, 2 m target and 3 m target. From left to right (within each set): 3.5 kHz emission, 4.0 kHz emission and 4.5 kHz emission. In the click recordings, the emission and echo are temporally separated, while for the noise recordings they temporally overlap due to the longer emission duration (500 ms). It can be seen that emissions with higher spectral frequency content lead to echoes with higher intensity. The abbreviation a.u. refers to ‘arbitrary units’.

For each type of emission (click, noise) 12 recordings were made (3 frequency ranges × 4 target conditions). In a first processing step, we applied a level correction to equate recorded sound intensity of the emission across the three spectral frequency conditions. Discrepancies in the recorded emissions arose because the beam pattern of the speaker generating the emissions differed across frequencies thus leading to lower intensity of higher frequency emissions measured at the ear. To avoid the possibility that differences in the intensity of the emission would bias processing of the subsequent echo via forward masking/echo suppression (Litovsky, Colburn, Yost, & Guzman, 1999; Wallach, Newman, & Rosenzweig, 1949), we matched the intensity of emissions across conditions. This was done by multiplying the 3.5 and 4.5 kHz emission recordings with scaling factors (i.e. single numerical values) in order to equate the peak intensity of the emission to that in the 4 kHz conditions (in the target-absent recordings). We define the term *peak intensity* as the maximum absolute recorded sound value. These scaling factors are shown in Table 2 and had been obtained based on the difference in peak intensity of recordings in the target absent conditions. Furthermore, a scaling factor of 1.128 was applied to the noise emission recordings to equate the intensity of the noise emission (without the echo present) to that of the click. The sounds following these alterations are shown in Figure 3. As can be seen in the images in Figure 3, although the intensity of the emission is matched in these recordings, the intensity of the echo increases with increasing spectral frequency of the emission. This effect is expected based on the fact that sound waves of higher frequencies are composed of shorter wavelengths, which lead to stronger echoes from an object of the size used here (see Apparatus and Setup).

**Table 2.** Scaling Factors Applied to the Recordings, Prior to Normalisation of Echo Intensity.

Clicks			Noise		
3.5 kHz	4.0 kHz	4.5 kHz	3.5 kHz	4.0 kHz	4.5 kHz
0.794	1.000	1.288	0.732	1.000	1.672

*Note.* These scaling factors were applied in order to address the discrepancies in the emissions recorded at the ear that arose from irregularities in the speaker’s beam pattern across different frequencies. The recordings of the 3.5 and 4.5 kHz clicks and noise were scaled in order to equate their peak intensity to that of the 4.0 kHz recordings.

**Figure 4.**
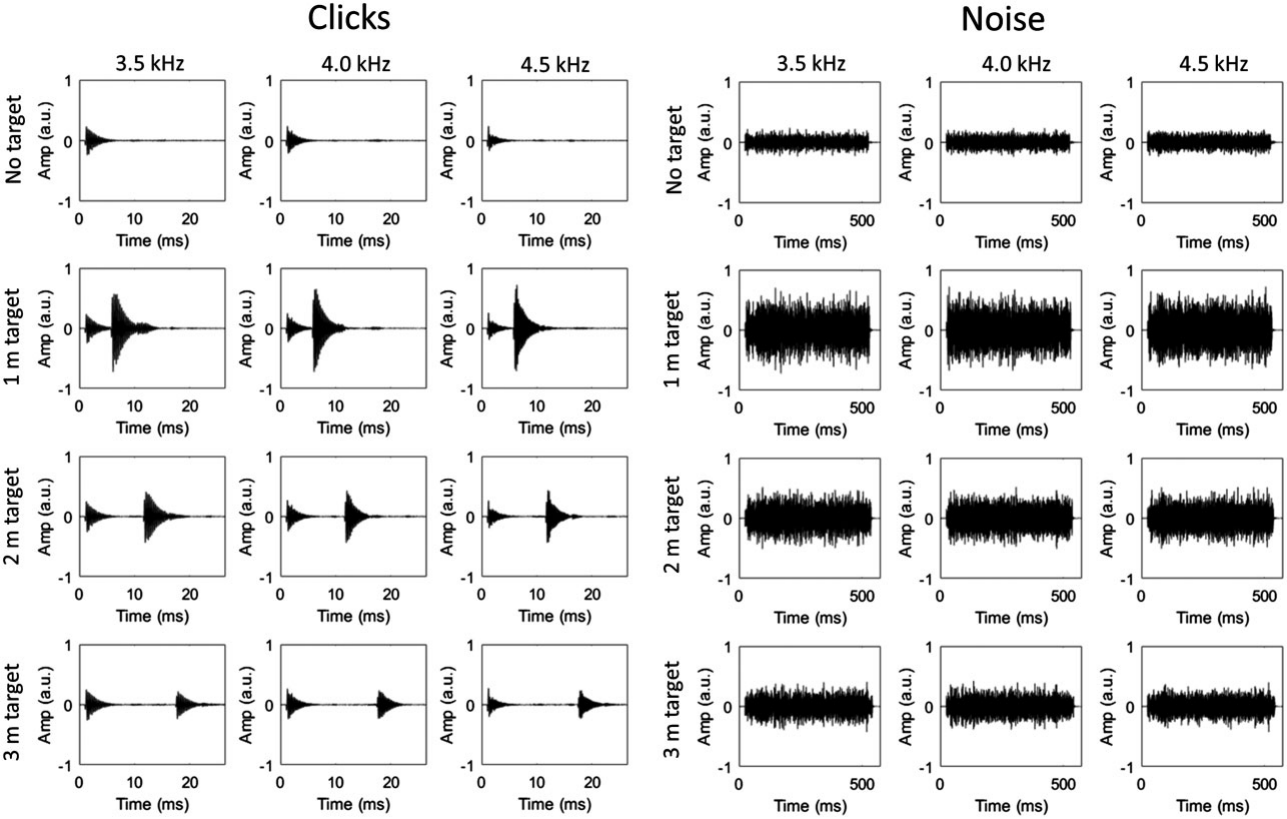
Waveforms of the recorded click and noise emissions that have been digitally altered to equate the intensity of the echoes across emission frequencies. The clicks are shown in the left set of images, and the noise in the right set. From top to bottom: no target, 1 m target, 2 m target and 3 m target. From left to right (within each set): 3.5 kHz emission, 4.0 kHz emission and 4.5 kHz emission. In the click recordings, the emission and echo are temporally separated, while for the noise recordings they temporally overlap due to the longer emission duration (500 ms). The intensity of the echoes for the 3.5 and 4.5 kHz emissions have been digitally altered to match that of the echo for the 4.0 kHz emission. The abbreviation a.u. refers to ‘arbitrary units’.

In order to test the hypothesis that this increase in echo intensity might underlie superior performance for emissions with higher spectral frequencies, we used these sounds to create a further set of stimuli in which the peak intensity of the echo had been equated across levels of spectral frequency (separately for each level of target distance). In order to do this, the temporal onset of the echo at each level of target distance had to be identified. This was done by visual inspection of the waveforms for the 4.0 kHz click recordings, with the point at which the waveform first rose above the noise floor being taken as the temporal onset of the echo. This point, taken by inspection of the recorded click waveforms at each level of target distance, was also taken as the onset point for the echo in the equivalent noise recordings. Any sound data following these identified time points in the recordings of the 3.5 kHz and 4.5 kHz emissions were then multiplied by respective scaling factors in order to equate their peak intensity to that in the recording of the 4.0 kHz emission. These scaling factors are shown in Table 3, and the scaled waveforms are shown in Figure 4. Thus, two sets of sound recordings were used in the experiment – one in which the peak intensity of the echoes differed and one in which the peak intensity of the echoes had been digitally equated.

**Table 3.** Scaling Factors Applied to Parts of the Recordings Containing the Echo in Order to Equate the Intensity of the Echo.

	Clicks			Noise		
Distance	3.5 kHz	4.0 kHz	4.5 kHz	3.5 kHz	4.0 kHz	4.5 kHz
1 m	1.315	1.000	0.792	1.282	1.000	0.788
2 m	1.280	1.000	0.897	1.364	1.000	0.880
3 m	1.315	1.000	0.758	1.270	1.000	0.727

*Note.* The recordings of the 3.5 and 4.5 kHz clicks and noise were scaled in order to equate the peak intensity of the echo to that of the 4.0 kHz recordings.

As an example, here is the step-by-step processing that was applied to the recorded 3.5 kHz click sounds. The peak intensities of the original 3.5 kHz and 4.0 k Hz click recordings with no object present were computed, where peak intensity refers to the maximum absolute sound value. Based on these values, all recordings of the 3.5 kHz clicks (i.e. target absent, 1 m target, 2 m target and 3 m target) were multiplied by 0.794 in order to bring the peak intensity of the 3.5 kHz click to that of the 4.0 kHz click. In order to create a second set of sound recordings, in which the peak intensity of the echo was equated across different values of the emission frequency, the peak intensity of the echo at each target distance was first calculated for both the 3.5 kHz and 4.0 kHz recordings. All recorded sound values after the initial onset of the echo in the 3.5 kHz click recordings were then multiplied by either 1.315 (target at 1 m), 1.280 (target at 2 m) or 1.315 (target at 3 m) in order to equate the peak intensity of the echo at 3.5 kHz to that at 4.0 kHz, but leaving the clicks unchanged.

### Behavioural Experiment

#### Participants

Twelve participants (8 women, 4 men; age range 19–41, *M* = 27.8 years) completed all components of the behavioural experiment. All participants reported having normal or corrected to normal vision and hearing, and reported no prior experience using echolocation. Sighted participants who are not familiar with echolocation have been successfully trained to a good level of performance in previous tasks of echolocation (e.g. Rowan et al., 2013, 2015; Schenkman & Nilsson, 2010, 2011; Tonelli et al., 2016). Participants were compensated either at a rate of £6/hour or with the equivalent participant pool credit.

#### Task and procedure

Participants were tested in the same sound-insulated and echo-acoustic dampened room in which the sound recordings had been made. Sounds were played to participants through binaural in-ear headphones (Etymotic Research ER4B MicroPro) driven by a Dell Latitude E7470 laptop (Intel Core i56300U CPU 2.40 GHz, 8 GB RAM, 64-bit Windows 7 Enterprise) through a USB Soundcard (Creative Sound Blaster X-Fi HD Sound Card; Creative Technology Ltd., Creative Labs Ireland, Dublin, Ireland). Sounds were played to participants at a level at which the highest peak intensity (the 4.5 kHz noise emissions with target present at 1 m, with echo intensity not equated) was presented at 80 dB SPL. Participants sat upright wearing a blindfold and gave their response using a keyboard.

Trials were presented in blocks that were defined by two factors: emission type (click, noise) and echo intensity (not equated, equated), and the order of these blocks was fully counterbalanced across participants. Six repetitions of each of these blocks were run across two separate testing sessions, with three repetitions in the first session and three in the second, and the two sessions were carried out on separate days. At the start of each session, participants completed a training block of 16 trials for each of the four block permutations. Each block contained 72 randomly presented trials of two factors: emission frequency (3.5, 4.0, 4.5 kHz in the ratio 1:1:1) and target condition (absent, 1, 2, 3 m in the ratio 3:1:1:1).

Participants pressed a key to initiate the onset of each trial and were then presented with a single sound (e.g. a 4.5 kHz click with target at 2 m with echo intensity not equated). After hearing the sound they then judged using a six-rating confidence scale (by pressing one of six keys on the keyboard) whether they heard the target object. The scale ranged from *very confident object absent* (1) to *very confident object present* (6), with intermediate responses to indicate less confident judgments. Participants received auditory feedback (50 ms tone) on each trial to indicate whether they were correct or not (1200 or 900 Hz tone, respectively), with a rating of 1 to 3 being classed as a correct response for target-absent trials and a rating of 4 to 6 being classed as a correct response for target-present trials. Participants were instructed to prioritise accuracy over speed in their response.

## Results

Participants’ performance in the click and noise conditions was analysed separately, and participants’ sensitivity to the target was calculated separately for each permutation of the following experimental conditions: echo intensity (equated, not equated), emission frequency (3.5, 4.0, 4.5 kHz) and target distance (1, 2, 3 m). Sensitivity was calculated by tabulating the number of responses for each of the six confidence levels for both target-absent trials and target-present trials, and using the software RScorePlus (Harvey, 2002) to fit a Gaussian unequal-variance signal detection model and derive a discriminability index (*d*_a_). *d*_a_ does not assume equal variance between the participants’ underlying *noise* and *signal + noise* Gaussian probability distributions, but is equivalent to *d*’ (*d*-prime) in the case of equal variance. A higher *d*_a_ indicates a greater sensitivity to the target, and a *d*_a_ of zero indicates no sensitivity. Table 4 shows the response probabilities (mean, minimum and maximum values across all 12 participants) for each of the six confidence responses that participants could give on a single trial. Although the probabilities are low for some of the response categories, all participants did use the full scale. Furthermore, a chi-square statistic was calculated for each of the fitted models, computed from the log likelihood of the fit, as an indication of each of the model’s goodness-of-fit. None of these statistics were significant (i.e. all *p* values > .05), indicating that the variance in the response data is well described by the fitted models.

**Table 4.** Responses Probabilities Statistics, Taken From All Participants and Stimulus Conditions.

	1 (highly confident target absent)	2 (slightly confident target absent)	3 (guessing target absent)	4 (guessing target present)	5 (slightly confident target present)	6 (very confident target present)
Mean	0.245	0.129	0.112	0.086	0.117	0.311
Min	0.006	0.023	0.007	0.006	0.031	0.023
Max	0.413	0.298	0.477	0.259	0.330	0.451

### Clicks

**Figure 5.**
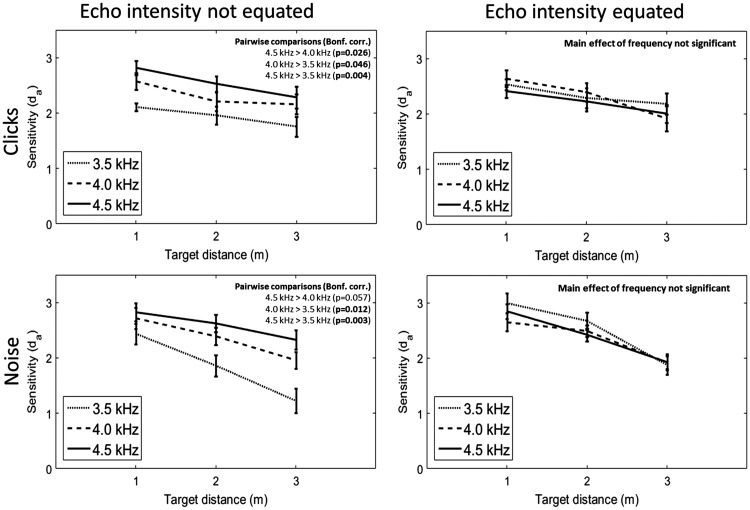
Results from the behavioural experiment. Participants’ performance in detecting the target when the intensity of the echoes was not equated is shown in the left panels, with click emissions represented in the upper panel and noise emissions in the lower panel. As can be seen, there was an advantage in using emissions (both clicks and noise) containing higher spectral frequencies (from 3.5 to 4.5 kHz). In contrast, participants’ performance in detecting the target when the intensity of the echoes was equated is shown in the right panels. In these conditions, there was no advantage for using clicks or noises containing higher spectral frequencies. Error bars represent the standard error of the mean, with between-subject variance removed.

Behavioural results for the click emissions are shown in Figure 5 (top two panels). An initial 2 (echo intensity: equated or not) × 3 (emission frequency: 3.5, 4, 4.5 kHz) × 3 (target distance: 1 m, 2 m, 3 m) repeated measures ANOVA was carried out, with *d*_a_ as the dependent variable. This revealed a significant interaction between echo intensity and emission frequency, *F*(2, 22) = 10.146, *p* = .001, η_p_^2 ^= 0.480), implying that clicks of different spectral frequencies affected performance differently depending on whether the intensity of the echoes was equated or not. To follow-up this interaction, two separate 3 (emission frequency: 3.5, 4, 4.5 kHz) × 3 (target distance: 1 m, 2 m, 3 m) repeated measures ANOVAs were carried out for conditions in which the echo intensity had been equated, or not.

When the echo intensity was not equated across levels of emission frequency, there was a significant effect of emission frequency, *F*(2, 22) = 13.338, *p* < .001, η_p_^2 ^= 0.548, with mean *d*_a_ values of 1.942, 2.313 and 2.543 for 3.5, 4.0 and 4.5 kHz clicks, respectively. This shows that participants’ sensitivity to the target was higher for clicks containing higher spectral frequencies. Pairwise comparisons across emission frequencies (with *p* values adjusted for multiple comparisons using the Bonferroni method) revealed that *d*_a_ was significantly higher for 4.5 kHz clicks compared to 4.0 kHz clicks (*p* = .026), for 4.5 kHz clicks compared to 3.5 kHz clicks (*p* = .004) and for 4.0 kHz clicks compared to 3.5 kHz clicks (*p* = .046). There was a significant effect of target distance, *F*(1.265, 13.913) = 6.639, *p* = .017, Greenhouse-Geisser corrected, η_p_^2 ^= 0.376, with mean *d*_a_ values of 2.500, 2.233 and 2.066, respectively, for target distances of 1, 2 and 3 m, respectively. This shows that participant’s sensitivity to the target decreased with target distance. None of the individual pairwise comparisons across target distances (Bonferroni corrected) were significant, however. There was no significant interaction between emission frequency and target distance, *F*(4, 44) = 0.946, *p* = .447, η_p_^2 ^= 0.079.

In comparison, when echo intensity was equated across levels of emission frequency, there was no significant effect of emission frequency, *F*(2, 22) = 0.900, *p* = .421, η_p_^2 ^= 0.076, with mean *d*_a_ values of 2.336, 2.317 and 2.216 for 3.5, 4.0 and 4.5 kHz clicks, respectively. This shows that the advantage for using clicks with higher spectral frequencies was not observed when the clicks with higher spectral frequencies did not result in more intense echoes. There was a significant effect of target distance, *F*(2, 22) = 7.769, *p* = .003, η_p_^2 ^= 0.414, with mean *d*_a_ values of 2.528, 2.304 and 2.036 for target distances of 1, 2 and 3 m, respectively. Again, this shows that participant’s sensitivity to the target decreased with target distance. Pairwise comparisons across target distances (Bonferroni corrected) showed that *d*_a_ was significantly higher (*p* = .014) only for the detection of 1 m targets compared to 3 m targets. There was no significant interaction between emission frequency and target distance, *F*(4, 44) = 2.277, *p* = .076, η_p_^2 ^= 0.171.

### Noise

Behavioural results for the noise emissions are shown in Figure 5 (bottom two panels). A 2 (echo intensity: equated or not) × 3 (emission frequency: 3.5, 4, 4.5 kHz) × 3 (target distance: 1 m, 2 m, 3 m) repeated measures ANOVA was carried out, with *d*_a_ as the dependent variable. This revealed a significant interaction between echo intensity and emission frequency, *F*(1.219, 13.405) = 18.898, *p* < .001, Greenhouse-Geisser corrected, η_p_^2 ^= 0.632, implying that noise of different spectral frequencies affected performance differently depending on whether the intensity of the echoes was equated or not. To follow up this interaction, two separate 3 (emission frequency: 3.5, 4, 4.5 kHz) × 3 (target distance: 1 m, 2 m, 3 m) repeated measures ANOVAs were carried out for conditions in which the echo intensity had been equated, or not.

When the echo intensity was not equated across levels of emission frequency, there was a significant effect of emission frequency, *F*(2, 22) = 15.885, *p* < .001 η_p_^2 ^= 0.591, with mean *d*_a_ values of 1.842, 2.355 and 2.592 for 3.5, 4.0 and 4.5 kHz noise, respectively. Pairwise comparisons across emission frequencies (Bonferroni corrected) revealed that *d*_a_ was significantly higher for 4.0 kHz noise compared to 3.5 kHz noise (*p* = .012) and for 4.5 kHz noise compared to 3.5 kHz noise (*p* = .003), but was not significantly higher for 4.5 kHz noise compared to 4.0 kHz noise (*p* = .057). There was a significant effect of target distance, *F*(1.276, 14.037) = 27.122, *p* < .001, Greenhouse-Geisser corrected, η_p_^2 ^= 0.711, with mean *d*_a_ values of 2.659, 2.292 and 1.837 for target distances of 1, 2 and 3 m, respectively. Again this shows that participant’s sensitivity to the target decreased with target distance. Pairwise comparisons across target distances (Bonferroni corrected) showed that *d*_a_ was significantly higher for 1 m targets compared to 2 m targets (*p* = .005), for 1 m targets compared to 3 m targets (*p* = .001) and for 2 m targets compared to 3 m targets (*p* = .001). There was a significant interaction between emission frequency and target distance, *F*(4, 44) = 4.946, *p* = .002, η_p_^2 ^= 0.310. The bottom left panel in Figure 5 illustrates that this interaction arose because performance differences across emission frequencies became more pronounced as target distance increased. This is also confirmed with three post-hoc ANOVAs, each assessing the effect of emission frequency at one of the three target distances (with *p* values adjusted for multiple comparisons using the Bonferroni method). There was no significant effect of emission frequency at a target distance of 1 m, *F*(2, 22) = 2.921, *p* = .225, η_p_^2 ^= 0.210, but there was a significant effect of emission frequency at a target distance of 2 m, *F*(2, 22) = 12.914, *p* < .001, η_p_^2 ^= 0.450, and at 3 m, *F*(2, 22) = 20.257, *p* < .001, η_p_^2 ^= 0.648.

In comparison, when echo intensity was equated across levels of emission frequency, there was no significant effect of emission frequency, *F*(2, 22) = 1.959, *p* = .165, η_p_^2 ^= 0.151, with 3.5 kHz clicks giving a mean *d*_a_ value of 2.516, 4.0 kHz a mean value of 2.354 and 4.5 kHz a mean of 2.400. This shows that the advantage for using noise with higher spectral frequencies was not observed when the noise with higher spectral frequencies did not result in more intense echoes. There was a significant effect of target distance, *F*(2, 22) = 36.763, *p* < .001, η_p_^2 ^= 0.770, with mean *d*_a_ values of 2.831, 2.530 and 1.190 for target distances of 1, 2 and 3 m, respectively. Again, this shows that participants’ sensitivity to the target decreased with target distance. Pairwise comparisons across target distances (Bonferroni corrected) showed that *d*_a_ was significantly higher for 1 m targets compared to 2 m targets (*p* = .044), for 1 m targets compared to 3 m targets (*p* < .001) and for 2 m targets compared to 3 m targets (*p* = .001). There was no significant interaction between emission frequency and target distance, *F*(4, 44) = 2.566, *p* = .051, η_p_^2 ^= 0.189.

## General Discussion

In this experiment, participants listened to pre-recorded sounds that contained an emission (either a click or noise) and judged whether they could hear an object that was present at a distance of either 1, 2 or 3 m, or absent altogether. Results showed that participants’ sensitivity to the target increased as the peak spectral frequency of the emission (both clicks and noise) increased from 3.5 to 4.5 kHz. This advantage was not present, however, when the sounds were digitally altered such that the intensity of the echoes was equated across emissions of different frequency ranges. We conclude, therefore, that emissions with higher spectral frequencies can benefit echolocation performance in conditions where they lead to an increase in echo intensity.

This is the first study to directly test whether emissions containing higher spectral frequencies confer an advantage in echolocation. A previous study did show that the spectral content of the emission was associated with echolocation performance (Thaler & Castillo-Serrano, 2016), but this was only correlational evidence and the result was driven by the performance of a small number of expert echolocators who, relative to the non-expert controls, produced clicks containing higher spectral frequencies and performed significantly better. There was also evidence in a small sample of echolocators that those who produced clicks containing higher spectral frequencies performed better in a task of angle discrimination (Teng et al., 2012; Thaler et al., 2017) but, again, this was only correlational evidence. In contrast, the design of the current study allowed us to show that participants’ ability to detect an object using echolocation improved when they used emissions containing higher spectral frequencies. Importantly, we included both clicks (lasting ∼ 5 ms) and noise bursts (lasting 500 ms) as emissions in our experiment, and the effect was the same for both emissions. With respect to the noise emissions, our results generally agree with those by Rowan et al. (2013, 2017). Specifically, in those studies participants were more accurate in detecting or localising an object in virtual acoustic space using high-pass (>3 kHz), compared to low-pass (<3 kHz), filtered noise. Yet, they also found that for object azimuth localization the addition of low frequency sound (i.e. use of broad band noise as compared to high pass noise only) impaired echo processing (Rowan et al., 2013), while for target detection the addition of low frequency sound actually improved performance (Rowan et al., 2017). Importantly, in our stimuli low frequency sound (<3 kHz) was always present. Thus, if any effects from low frequency sound occurred they would have been the same across conditions. As such, our results clearly highlight the importance of high frequency emissions due to higher echo intensity, above and beyond the effects of low frequency sound as described by Rowan et al. (2013, 2017).

It should be noted, however, that producing emissions of higher spectral frequencies may not confer an advantage in all situations. Flanagin et al. (2016), for instance, found that participants’ ability to estimate the size of a virtual room did not correlate with the spectral content of their clicks. This makes sense, however, because emissions of higher spectral frequencies will only increase echo intensity when echolocating an object of finite size, but not when echolocating an enclosed space. Our current findings are therefore consistent with what Flanagin et al. (2016) found.

It is important to address whether the results of the present study can be generalised to the use of echolocation in a more ecologically valid setting. Importantly, we used emissions with peak frequencies between 3.5 and 4.5 kHz – a range that includes frequencies contained in natural human mouth clicks of expert echolocators (Thaler et al., 2017; Zhang et al., 2017). Furthermore, the click emissions we used were similar to those that people make (Thaler et al., 2017; Zhang et al., 2017. Also, the object size we used was relevant to people who use echolocation in everyday life (e.g. to detect side panel of a bus shelter, a large tree or a person). In sum, it is reasonable to assume that our findings generalise to natural human echolocation. It was a necessity in the design of this study, however, that participants did not actively generate their own emissions, as otherwise we would have lacked control over acoustics of emissions. It has been shown in a previous study (Thaler & Castillo-Serrano, 2016), however, that when expert echolocators detect a target of the same size used here and at distances also used here, there is no difference in their performance when they create their own emissions compared to when they use artificial ones similar to those used here. We expect, therefore, that the current results generalise to active echolocation.

It is important to address whether the use of feedback tones in our experiment could limit the generalisability of our results. The positive/negative feedback tones were used as a proxy for the real feedback that echolocators would receive in a realistic setting, whose perceptual judgments would be positively or negatively reinforced based on their accuracy (e.g. physical feedback from touching objects, or from colliding with an undetected obstacle). The use of feedback in our task, therefore, was not entirely arbitrary, but we do expect that it accelerated participants’ learning of echolocation.

An additional point to address is whether participants did in fact use ‘true’ echolocation to solve this task, or whether they instead relied on a heuristic based on a simple acoustic property (e.g. a judgment based purely on loudness or pitch). Two aspects of the design of our experiment make the use of such a heuristic unlikely. First, participants made a judgment only on a single sound on each trial, as opposed to an alternative design in which they would identify which of two sounds contained an echo. Such an alternative design might conceivably allow participants to use a heuristic such as ‘Choose the sound that is loudest’, but it would be difficult to apply such a heuristic in our single-interval-trial design as there are no two sounds to compare. Second, the random presentation of trials in the present study ensured that both the spectral content of the sound as well as the target distance were unpredictable from trial to trial. The overall perceived loudness and pitch of the echo, therefore, were unpredictable to participants, making it difficult for them to rely on a simple heuristic to detect the object.

To conclude, using emissions containing higher spectral frequencies improved echolocation performance in an object-detection task. Importantly, this was true for both click and noise emissions, and generalised to different target distances. Emissions containing higher spectral frequencies induced louder echoes, but when sound intensity was equated across emissions of different frequency ranges participants no longer showed an advantage in using the higher frequency emissions. This shows that emissions with higher spectral content can benefit echolocation performance in conditions where they lead to an increase in echo intensity. The findings suggest that people who train to echolocate should be instructed to make emissions (e.g. mouth clicks) with higher spectral frequency content.
